# Xanthogranulomatous Salpingo-Oophoritis Presenting as an Ovarian Malignancy

**DOI:** 10.7759/cureus.13363

**Published:** 2021-02-15

**Authors:** Tara Manandhar, Sanyukta Rajbhandari, Achala Thakur, Sangeeta Bhandari, Sushil Dhakal

**Affiliations:** 1 Obstetrics and Gynaecology, B.P. Koirala Institute of Health Sciences, Dharan, NPL; 2 Obstetrics and Gynaecology, Maya Metro Hospital, Dhangadhi, NPL; 3 Pathology, Maya Metro Hospital, Dhangadhi, NPL

**Keywords:** xanthogranulomatous, salpingitis, oophoritis, hysterectomy

## Abstract

Xanthogranulomatous salpingo-oophoritis is an extremely rare entity. The clinical features are similar to the common benign and malignant adnexal diseases, making it difficult to diagnose. Here we present a case of pelvic mass with high level of tumor markers who was operated with suspicion of adnexal tumor. Histopathology revealed it to be a case of xanthogranulomatous salpingo-oophoritis.

## Introduction

Xanthogranulomatous inflammation is an uncommon cause of chronic inflammation, which leads to the destruction of the normal tissue of the affected organ. It is characterized by the accumulation of lipid-laden foamy macrophages intermixed with neutrophils, lymphocytes, and plasma cells along with multinucleated giant cells [1]. The kidney is the most commonly affected organ by xanthogranulomatous inflammation but it may also affect various organs such as the gallbladder, stomach, anorectal area, bone, urinary bladder, testis, and epididymis. Xanthogranulomatous inflammation affecting the female genital tract is extremely rare, with only a handful of cases been reported, usually involving the fallopian tube and ovaries [2]. The cases usually present as a pelvic inflammatory disease that does not respond to antibiotics and/or as a mass lesion mimicking pelvic malignancy [3]. Here we present a case of xanthogranulomatous salpingo-oophoritis presenting as an ovarian malignancy.

## Case presentation

A 68-year postmenopausal woman, P8L5CD3 (pregnancies eight, out of which five living and three child deaths), was admitted with chief complaints of intermittent lower abdominal pain along with a sensation of incomplete voiding for the last two months. There were no other complaints. She was a chronic smoker with a history of chronic obstructive pulmonary disease. She was hemodynamically stable. The abdomen was soft, scaphoid, and non-tender, and there was no palpable mass. The cervix was atrophied and taken up by the vaginal wall with no clear distinction between them (cervix flushed with the vagina), and there was keratinization on the posterior vaginal wall on per speculum examination. On per vaginal examination, the uterus was atrophied. There was an ill-defined hard mass in the pouch of Douglas, measuring around 8×8 cm^2^, fixed to the surrounding structure and was non-tender.

Ultrasonography (USG) showed bilateral thick-walled adnexal cyst (right measuring 4×4 cm^2^ and left measuring 4×2 cm^2^) with internal septation, a bulky uterus, few echogenic foci within the endometrial cavity, thickening of the urinary bladder wall, and left renal simple cortical cyst. The contrast-enhanced computed tomography of the abdomen and pelvis was planned but refused by the patient due to financial constraints. Her complete blood count was within a normal range. Alkaline phosphatase was 864 IU/L, cancer antigen 125 (CA-125) was 142.5 U/mL (high), and carcinoembryonic antigen (CEA) was 4.5 mg/mL.

A staging laparotomy was performed, which revealed an atrophic uterus. There was a mass measuring 6×6 cm^2^ on the right adnexa, which was densely adhered to the omentum and large bowel. Anteriorly, the lesion had adhered to the urinary bladder with degenerated broad ligament fibroid. Bilateral fallopian tubes and ovaries seemed to be normal. The patient underwent a total abdominal hysterectomy with bilateral salpingo-oophorectomy. The patient had an uneventful postoperative recovery and was discharged on the sixth postoperative day. On follow-up, the patient was examined clinically, transabdominal USG was performed, and the CA-125 value was assessed. At the one- and three-month follow-up, the patient was asymptomatic and her CA-125 value was within the normal limit. 

**Figure 1:**
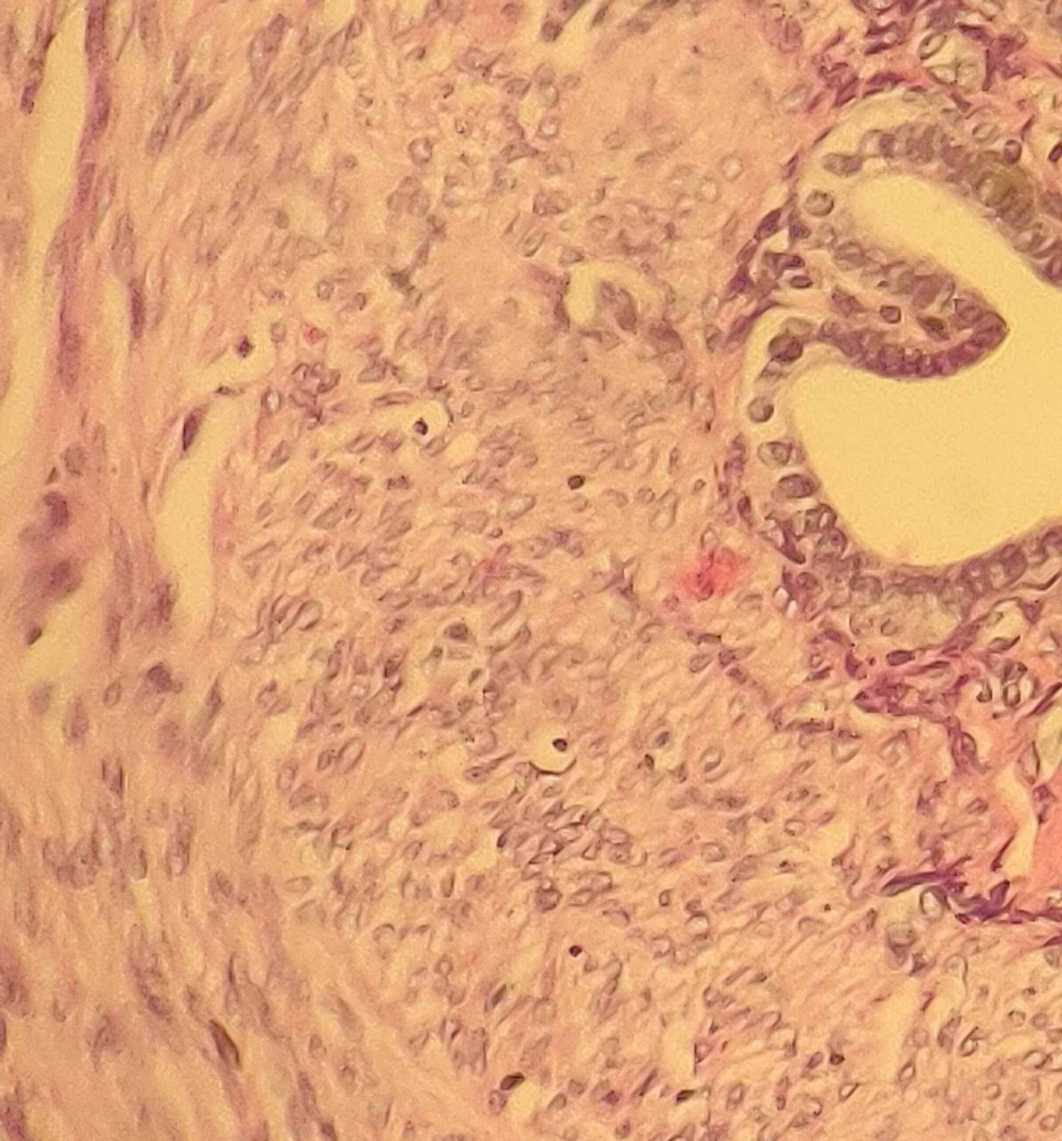
Histopathological examination of fallopian tube showing acute and chronic inflammatory cells, with the tube wall containing predominantly foamy macrophages.

**Figure 2:**
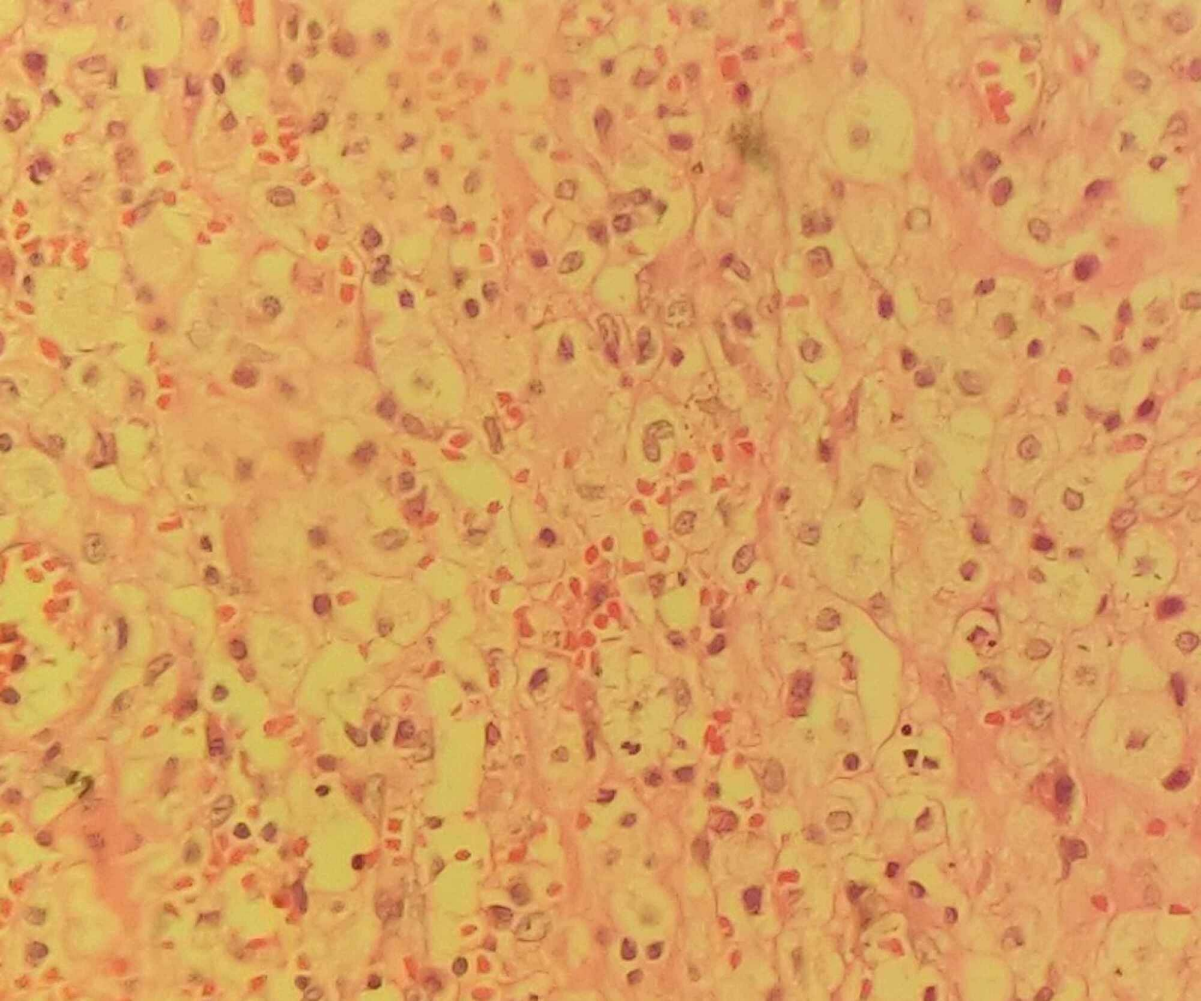
Histopathological examination of the ovaries showing foamy macrophages admixed with lymphocytes and neutrophils.

Histopathological examination of her fallopian tube revealed flattened plica. The lumen contained acute and chronic inflammatory cells. The wall of the tube contained predominantly foamy macrophages admixed with lymphocytes and occasional neutrophils (Figure 1). Histopathological examination of the ovaries revealed ovarian parenchyma infiltrated by inflammatory cells consisting of macrophages, with most of them being foamy types with admixed lymphocytes and neutrophils (Figure 2).

## Discussion

Xanthogranulomatous inflammation is a chronic destructive inflammation of the affected organ. Histologically there is a presence of a large number of lipid-laden foamy macrophages intermixed with neutrophils, lymphocytes, and plasma cells, which is a characteristic of xanthogranulomatous inflammation with or without multinucleated giant cells (Touton giant cells) [1]. Xanthogranulomatous inflammation in the female genital tract mainly affects one side and has different terminologies such as xanthogranulomatous salpingitis and ovarian fibro xanthoma [3,4]. However, it is less common in the female genital tract with only a handful of cases been reported. There were 13 cases of xanthogranulomatous salpingitis in the years 2003 to 2018, as reported by Chiesa-Vottero, in which most of the clinical presentations mimicked female genital malignancy [2]. Xanthogranulomatous salpingitis has been found in patients aged 21-75 years with a mean age of 45 years [2,5]. The youngest age at which it was detected was at the age of two years, as reported by Tanwar et al. [6]. The exact etiology of the xanthogranulomatous salpingitis is not clear but it is associated with the conditions that produce the foam cells. Pelvic inflammatory disease, endometriosis, ineffective and unsuccessful antibiotic therapy, abnormality in lipid metabolism, intrauterine contraceptive device, radiotherapy, and ineffective clearance of bacteria (*Escherichia coli, Proteus spp., Staphylococcus aureus, Bacteroides fragilis*, and *Salmonella typhi*) by phagocytes are some conditions where xanthogranulomatous inflammation was seen [1,7,8].

The clinical scenario varies widely between the patients. Patients may present with pain in the lower abdomen, fever, dysmenorrhea, dyspareunia, chronic pelvic pain, bleeding, poor appetite, mass in the abdomen, and/or infertility [3-5,9]. The destructive and mass-forming nature of the disease makes it hard, if not impossible, to distinguish it from malignancy both clinically and radiologically. In our study, the patient presented with the adnexal mass and a raised CA-125, mimicking the pelvic tumor, and was operated with the suspicion of malignancy. However, it was an intraoperative surprise with an inflammatory adnexal mass and minimal changes in the fallopian tubes and ovaries. Histopathological examination clinched the diagnosis of xanthogranulomatous salpingo-oophoritis. As seen in our case, the disease is a clinical challenge, and the xanthogranulomatous inflammation is found in a histopathological report of a patient who had undergone a hysterectomy for suspected endometrial malignancy, ovarian malignancy, endometriosis, recurrent pyometra, and large uterine myoma [2,4,5,7]. Therefore, being a rare disease and its clinical features similar to the pelvic organ tumor, xanthogranulomatous salpingo-oophoritis is very hard to diagnose preoperatively and is usually revealed after histopathological examination. Thus, careful considerations are warranted to diagnose the origin of the inflammatory diseases and to appropriately treat the diseases in the female genital tract [5].

## Conclusions

Xanthogranulomatous salpingo-oophoritis is a very rare disease and presents with clinical symptoms similar to that of common benign and malignant adnexal disease and hence requires a high index of suspicion for diagnosis.
